# Interspecies Communication between Pathogens and Immune Cells via Bacterial Membrane Vesicles

**DOI:** 10.3389/fcell.2016.00125

**Published:** 2016-11-11

**Authors:** Katerina S. Jurkoshek, Ying Wang, Jaffre J. Athman, Marian R. Barton, Pamela A. Wearsch

**Affiliations:** Department of Pathology, Case Western Reserve UniversityCleveland, OH, USA

**Keywords:** extracellular vesicle, membrane vesicle, PAMPs, immunomodulation, *Mycobacterium tuberculosis*

## Abstract

The production of extracellular vesicles is a universal mechanism for intercellular communication that is conserved across kingdoms. Prokaryotes secrete 50–250 nm membrane vesicles (MVs) in a manner that is regulated by environmental stress and is thought to promote survival. Since many types of host-derived stress are encountered during infection, this implies an important role for MV secretion in bacterial pathogenesis. Accordingly, MVs produced by gram-positive and gram-negative pathogens contain toxins, virulence factors, and other molecules that promote survival in the host. However, recent studies have also shown that bacterial MVs are enriched for molecules that stimulate innate and adaptive immune responses. As an example, MVs may serve multiple, important roles in regulating the host response to *Mycobacterium tuberculosis* (Mtb), an intracellular pathogen that infects lung macrophages and resides within modified phagosomes. Previously, we demonstrated that Mtb secretes MVs during infection that may modulate infected and uninfected immune cells. Our present data demonstrates that Mtb MVs inhibit the functions of macrophages and T cells, but promote Major Histocompatibility Complex (MHC) class II antigen presentation by dendritic cells. We conclude that bacterial MVs serve dual and opposing roles in the activation of and defense against host immune responses to Mtb and other bacterial pathogens. We also propose that MV secretion is a central mechanism for interspecies communication between bacteria and host cells during infection.

## Introduction

Extracellular vesicles have been recognized in recent years as a novel form of intercellular communication that is conserved across species. Perhaps the most intriguing trend in the field is the growing number of reports of extracellular vesicle secretion by prokaryotes (Kim et al., 2015). For simplicity, we will refer to extracellular vesicles produced by bacteria as membrane vesicles (MVs). The composition and functions of MVs vary across species, but collectively suggest that MV secretion is a conserved pathway that promotes bacterial survival. Pathogenic bacteria secrete toxins and virulence factors via MVs, as well as factors that modulate or defend against host immune responses. Based on these findings, we propose that MV secretion facilitates the survival of bacterial pathogens in the host microenvironment, and therefore, is essential for virulence. In turn, we propose that innate and adaptive immune responses have evolved to respond to MV-associated cargo as a readily accessible source of PAMPs (pathogen-associated molecular patterns) and antigens. The article will discuss how MVs, such as those secreted by *Mycobacterium tuberculosis* (Mtb), participate in intercellular communication between pathogenic bacteria and the host immune system.

## The basics: biogenesis and functions of bacterial membrane vesicles

The first reports of vesicle secretion by bacteria were published in the 1960's, but the field has developed substantially in recent years (Kim et al., 2015). This trend was likely fueled by expanding interest in exosomes, a type of vesicle released by mammalian cells, and the recently discovered role of extracellular vesicles in intercellular communication. Bacterial MVs, which are 20–300 nm in size, serve as both a secretion and transport system for proteins, lipids, and nucleic acids. Thus, the functions of MVs derive from their molecular cargo. Accordingly, the first phase of research has focused on the purification and analysis of MVs produced by bacteria in axenic (i.e., single species) culture, and hundreds of such studies have established that many different types of bacteria secrete MVs. This includes gram-negative and gram-positive bacteria as well as pathogenic and non-pathogenic bacteria (Brown et al., 2015; Schwechheimer and Kuehn, 2015). Furthermore, MVs can transport their cargo to recipient cells of the same species, other bacterial species, or eukaryotic cells. Current evidence suggests diverse and important roles for bacterial MVs including, but limited to, nutrient uptake, antimicrobial defense, horizontal gene transfer, biofilm nucleation, and the trafficking of microbial products such as virulence factors and toxins during infection (Kim et al., 2015).

Our current understanding of bacterial MV biogenesis is limited. MVs released by gram-positive and *Mycobacterium* species are thought to derive from the plasma membrane, but the mechanism is unknown (Brown et al., 2015). MVs secreted by gram-negative bacteria are enriched for LPS (lipopolysaccharide) and outer membrane proteins indicating that vesicle budding proceeds from the outer membrane (Schwechheimer and Kuehn, 2015). Several models have been proposed for MV production by gram-negative bacteria, suggesting that vesicle biogenesis is regulated by multiple pathways. The structure or accumulation of LPS and lipids in the outer membrane are thought to contribute to membrane curvature and vesicle budding (Kadurugamuwa and Beveridge, 1995; Haurat et al., 2011; Elhenawy et al., 2016; Roier et al., 2016). In addition, disruption of crosslinks between the outer membrane and peptidoglycan layer promote MV release (Deatherage et al., 2009; Schwechheimer et al., 2014, 2015). Recently, a high-throughput screen of a whole genome *E. coli* knockout library identified 171 genes that affect vesicle biogenesis (Kulp et al., 2015). Interestingly, mutants of oxidative stress response pathways correlated with a hypovesiculation phenotype. This finding is consistent with the observation that MV production is upregulated in response to oxidative stress (Sabra et al., 2003; MacDonald and Kuehn, 2013; van de Waterbeemd et al., 2013). MVs are secreted constitutively by bacteria, but this process is also induced by iron-deprivation, antimicrobial peptides, and envelope stress (Manning and Kuehn, 2011; MacDonald and Kuehn, 2013; Prados-Rosales et al., 2014b). Taken together, current evidence suggests that MV biosynthesis promotes bacterial survival and adaptation to environmental stress.

## MV secretion at the host-pathogen interface

The success of a pathogen depends on its ability to survive and replicate within the host environment. For this purpose, bacterial pathogens have evolved mechanisms to acquire nutrients, defend against host responses, and in some cases, inflict damage upon host cells. Therefore, one would predict that pathogenic bacteria employ MVs to respond and adapt to host environment-derived stress. Several lines of experimental evidence support this conclusion. First, MV secretion by pathogenic bacteria is induced by conditions that are experienced during infection such as reactive oxygen species, iron-deprivation, and antimicrobial peptides (Manning and Kuehn, 2011; MacDonald and Kuehn, 2013; Prados-Rosales et al., 2014b). Of note, these studies employed axenic cultures and growth medium that mimicked host conditions during infection. Second, molecules that are secreted via MVs promote bacterial survival. For example, Mtb MVs contain the siderophore mycobactin and support growth in iron-deficient medium (Prados-Rosales et al., 2014b). Third, it is widely established that virulence factors and toxins are secreted via MVs. Examples include heat-labile enterotoxin (Enterotoxigenic *E. coli*), anthrax toxin (*Bacillus anthracis*), cholera toxin (*Vibrio cholerae*), listeriolysin O (*Listeria monocytogenes*), and alpha-hemolysin (*Staphylococcus aureus*) (Horstman and Kuehn, 2000; Rivera et al., 2010; Chatterjee and Chaudhuri, 2011; Lee et al., 2013). Collectively, these findings indicate that MV secretion is likely essential for bacterial virulence.

The functional advantages of MV-mediated secretion include the sorting, concentration, protection (within the vesicle lumen), and selective targeting of cargo. MV-mediated secretion also provides an attractive mechanism to explain how lipids and membrane proteins are released by bacteria or how pore-forming toxins traffic to host membranes. Thus, MVs may orchestrate the trafficking of microbial molecules to specific locations or host targets during bacterial infection. The secretion of virulence factors and toxins within MVs has been covered in depth by several recent reviews (Manning and Kuehn, 2013; Brown et al., 2015; Kim et al., 2015), so the remainder of this article will focus on the role of MVs in intercellular communication between bacterial pathogens and host immune cells.

## Immune modulation by bacterial membrane vesicles

The trafficking of microbial components via MVs is not only pathogen-beneficial. Innate immune responses are directed toward molecules that are shared by classes of pathogens such as gram-negative or gram-positive bacteria. More commonly known as PAMPs, these molecules are ligands for pattern recognition receptors (PRRs), activate immune cells, and elicit the production of pro-inflammatory cytokines (Kumar et al., 2011). MVs are a rich source of PAMPs, such as bacterial lipids, proteins, and nucleic acids (Kaparakis-Liaskos and Ferrero, 2015). As a primary example, LPS is a potent agonist of TLR4 and a component of the gram-negative outer membrane. Although TLR4 studies have employed purified LPS for nearly two decades, the physiological mechanism of LPS secretion is now known to involve MVs. This finding has been reported for many species of gram-negative bacteria including *Pseudomonas aeruginosa, Escherichia coli, Neisseria meningitidis*, and *Haemophilus influenzae* (Wispelwey et al., 1989; Gu and Tsai, 1991; Söderblom et al., 2005; Ellis et al., 2010). MVs produced by various bacterial species can also activate TLR2 or TLR5, two other surface PRRs that recognize bacterial lipoproteins and flagellin, respectively (Bergman et al., 2005; Durand et al., 2009; Ellis et al., 2010; Cecil et al., 2016; Rappazzo et al., 2016). In contrast, the response of TLR7, TLR8, and TLR9 to MV-associated nucleic acids appears to be weak or absent (Ellis et al., 2010; Cecil et al., 2016). This raises the intriguing possibility that cell surface TLRs, but not endosomal TLRs, evolved to recognize bacterial MVs and execute an immediate response to infection. Other PRRs that respond to extracellular bacteria are localized in the cytosol, but their discovery prompted the question of how and why bacterial products translocate to the host cell cytosol. An explanation was provided by the demonstration that MVs produced by *Helicobacter pylori, Pseudomonas aeruginosa, Neisseria gonorrhea, and Aggregatibacter actinomycetemcomitans* contain peptidoglycan, fuse with host cell membranes, and activate NOD1/NOD2 (Kaparakis et al., 2010; Thay et al., 2014). More recently, it was demonstrated that enterohemorrhagic *E. coli* MVs traffic LPS to the cytosol of host cells and activate non-canonical inflammasome signaling (Vanaja et al., 2016). In conclusion, vesicle-mediated transport provides a mechanism for the secretion of membrane-associated PAMPs as well as the delivery of bacterial components to cytosolic PRRs. PAMPs are typically indispensible to ensure detection by the innate immune system, consistent with the fact that MV secretion by bacteria is ubiquitous and likely essential for growth.

MVs secreted by bacterial pathogens also traffic antigens and stimulate adaptive immune responses. For example, MVs produced by *Neisseria* sp., *Salmonella typhimurium, Haemophilus influenza, Clostridium perfringens, and Vibrio cholerae* elicit antibody and/ or T cell responses when administered to mice (Bergman et al., 2005; Alaniz et al., 2007; Schild et al., 2009; Roier et al., 2012; Jiang et al., 2014). In some cases vaccination with MVs confers protective immunity to subsequent challenges (Alaniz et al., 2007; Holst et al., 2009; Roier et al., 2012). The antigenicity of MVs is also highlighted by the recent approval of Bexsero®, a *N. meningitidis* MV-based vaccine, in Europe and the United States for the prevention of serogroup B meningococcal disease (Gorringe and Pajon, 2012; Vernikos and Medini, 2014). Current evidence therefore suggests that MVs are a significant source of antigens during bacterial infection. However, this is a relatively understudied area and major questions remain. In general, MV-associated antigens are poorly characterized. It will be important to identify the immunodominant antigens in MVs and determine if MVs are the primary mechanism for their secretion. Second, antigen trafficking via MVs has not been broadly characterized for bacterial pathogens. Thus, it is currently unknown if MVs play a broad role or limited role in adaptive immune responses during infection.

To further complicate the picture, there is evidence for immune evasion by pathogen-derived MVs as well as immune modulation by commensal-derived MVs (Vidakovics et al., 2010; Shen et al., 2012; Waller et al., 2016). Nonetheless, our current knowledge collectively suggests that (1) MVs are a central mechanism for intercellular communication between bacteria and host cells during infection, and (2) MV secretion can be beneficial to the host as well as the pathogen. In other words, the biochemical composition of MVs is different for each species and determines whether MV secretion promotes bacterial virulence, host immunity, or both. Thus, the “net effect” of MV secretion on disease pathogenesis must be assessed on a case-by-case basis for each pathogen.

## *Mycobacterium tuberculosis* membrane vesicles

MV secretion by *Mycobacterium tuberculosis* (Mtb) is an excellent example of how MVs may modulate the host immune response in both a positive and negative manner. Mtb, the causative agent of tuberculosis, is an intracellular pathogen that infects lung macrophages and establishes latent infection for the lifetime of the host (O'Garra et al., 2013). To create a niche for survival, Mtb secretes a multitude of factors that inhibit macrophage effector functions or suppress host immune responses. For example, secreted virulence factors inhibit phago-lysosome fusion, allowing Mtb to reside within modified phagosomes. Another immune evasion mechanism involves the secretion of cell wall-derived TLR2 agonists. Although the activation of TLRs typically promotes immunity, prolonged TLR2 signaling during Mtb infection leads to the production of immunosuppressive cytokines (e.g., IL-10) and the inhibition of MHC-II antigen presentation (Harding and Boom, 2010; Richardson et al., 2015). As a result, the host mounts CD4+ T cell responses that are sufficient to contain Mtb bacilli within granulomas, but not eliminate the pathogen. MVs likely serve an important role in this balance of immunity and immune evasion that is characteristic of latent Mtb infection.

It was reported in 2011 that pathogenic and non-pathogenic mycobacteria produce MVs in axenic culture (Prados-Rosales et al., 2011). Proteomic analysis of Mtb MVs identified 48 proteins and a notable enrichment of lipoproteins which are potent agonists of TLR2 (Prados-Rosales et al., 2011). Lipid analysis demonstrated an enrichment of polar lipids, suggesting that MVs derive from the plasma membrane. MVs also contained Mtb glycolipids such as phosphatidylinositol mannosides (PIMs) and lipoarabinomannan (LAM). PIMs are TLR2 agonists (Harding and Boom, 2010), and LAM inhibits phago-lysosome fusion (Fratti et al., 2003). It had been known for many years that glycolipids and lipoproteins were secreted by Mtb bacilli, but the molecular mechanism was unknown. Thus, the discovery of MVs provided a rational explanation for the release of membrane-associated components from the Mtb cell wall. More recently, a comprehensive proteomic analysis identified 287 proteins in Mtb MVs, confirmed the enrichment of lipoproteins, and discovered dozens of novel MV-associated factors that impact Mtb pathogenesis (Lee et al., 2015). These include superoxide dismutase and catalase (virulence factors involved in protection from oxidative stress) as well as Ag85 and CFP10 (virulence factors and immunodominant T cell antigens). Aside from lipoproteins, however, the association of these proteins with Mtb MVs has yet to be validated in subsequent biochemical or functional studies.

To date, the function of Mtb MVs has largely focused on the trafficking of TLR2 agonists to immune cells. MVs that were purified from Mtb axenic cultures and then administered to macrophages *in vitro* stimulated TLR2-dependent production of TNFα, IL-1β, IL-6, and IL-12 (Prados-Rosales et al., 2011). Furthermore, intratracheal administration of purified Mtb MVs caused profound inflammation in the lungs of WT, but not TLR2-deficient mice. In accordance with these findings, the *virR* (vesiculogenesis and immune response regulator) Mtb deletion strain displays a hypervesiculation phenotype and elicits increased TLR2-dependent production of TNFα and IL-12 during macrophage infection (Rath et al., 2013). Taken together, these studies strongly implicate a role for MVs in the trafficking of PAMPs to immune cells.

**Figure 1:**
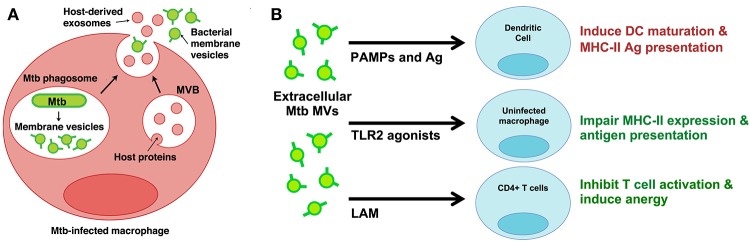
**Mtb MVs are secreted during infection and may modulate the functions of infected and uninfected immune cells. (A)** Proposed model for the trafficking of Mtb MVs during macrophage infection (Athman et al., 2015). MVs are secreted by Mtb bacilli within the phagosome; these vesicles are predicted to suppress macrophage effector functions and promote intracellular Mtb survival. In addition, Mtb MVs are released (mechanism unknown) into the extracellular environment and traffic to uninfected immune cells. **(B)** Potential mechanisms for the modulation of uninfected immune cells. Extracellular Mtb MVs may positively regulate the immune response by trafficking PAMPs and antigens to DCs, thereby promoting antigen presentation and T cell priming in the lymph node. In contrast, Mtb MVs are predicted to impair MHC-II expression and antigen presentation by uninfected macrophages as well as inhibit CD4+ T cell activation in the lung. Illustration modified from Athman et al. (2015), *The Journal of Immunology*. Copyright © 2015, The American Association of Immunologists, Inc.

Very few studies have developed infection models to study MV production by bacterial pathogens in the host environment. While investigating the properties of extracellular vesicles released from Mtb-infected macrophages, however, we discovered two distinct vesicle subsets—one that contained classic markers of exosomes and another that contained Mtb lipoproteins and glycolipids (Athman et al., 2015). We separated the two populations using density gradients and only the vesicles bearing Mtb molecules stimulated TLR2-dependent cytokine responses by uninfected cells. Based on similarities to vesicles produced by Mtb axenic cultures (Prados-Rosales et al., 2011), we concluded that Mtb secretes MVs within the phagosome of infected cells (Figure 1A). In support of this conclusion, MV secretion could be visualized by electron microscopy for Mtb bacilli recovered post-infection. Based on our findings we also concluded that Mtb MVs escape from infected macrophages into the extracellular environment (Figure 1A). Our work provides direct evidence for the production of MVs during Mtb infection and implies that MVs may traffic Mtb molecules to both infected and uninfected immune cells.

### Roles for Mtb MVs within infected macrophages

We propose the following model based on our current knowledge of MVs produced by Mtb and other pathogens. First, we predict that MV secretion is upregulated in response to host-derived stress, such as reduced pH or free radicals, upon Mtb entry into phagosomes. Once released from Mtb bacilli, MVs traffic virulence factors to host targets, thereby suppressing macrophage effector functions and promoting Mtb survival. For example, MVs may traffic LAM to the phagosomal membrane in order to block lysosome fusion or traffic lipoproteins to TLR2 in order to inhibit MHC-II antigen presentation and induce IL-10 production. Mycobactin released within MVs may participate in iron acquisition during chronic stages of Mtb infection. Thus, the roles for MVs within Mtb-infected cells are predicted to be pathogen-beneficial. Mtb strains with a null or hypovesiculation phenotype are needed to definitively connect MV secretion with the proposed functions, but are currently unavailable.

### Roles for Mtb MVs in the trafficking of microbial components to uninfected immune cells

**Figure 2:**
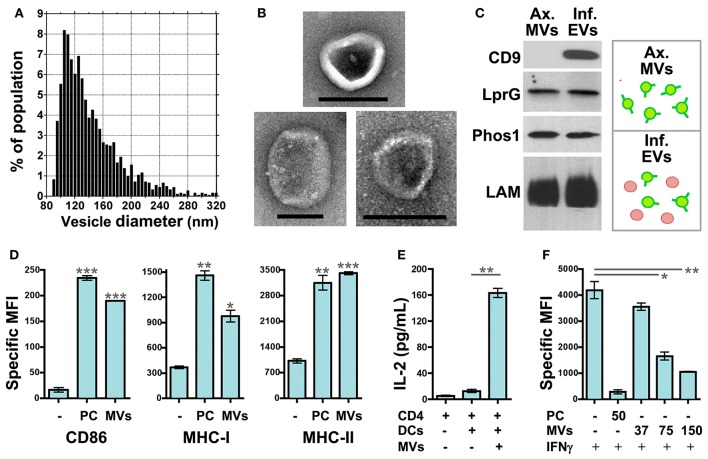
**Mtb MVs regulate the functions of uninfected immune cells in host- and pathogen-beneficial manners**. *MV purification*: Axenic Mtb cultures were grown in Sauton's broth, a defined, minimal medium, to late-log phase. MVs were purified from the conditioned medium by differential ultracentrifugation and gel filtration chromatography. First, the conditioned medium was centrifuged sequentially at 1000 × *g* and 3000 × *g* for 20 min each to remove cells and debris. The clarified medium was then passed through a 0.45 micron PVDF filter. After filtration, the medium was centrifuged for 2 h at 100, 000 × *g* using the Beckman Ti50.2 rotor. The supernatant was discarded, and the vesicle pellet was resuspended in PBS. Vesicles were further purified using IZON qEV size exclusion columns according to the manufacturer's instructions. **(A)** The size distribution of purified Mtb MVs was determined by TRPS (tunable resistive pulse sensing) using the IZON qNano system. Instrument settings and data analysis were performed as described (Athman et al., 2015). **(B)** Whole mount electron microscopy of Mtb MVs was performed as described (Athman et al., 2015). Note the typical cup-like morphology of extracellular vesicles. Scale bar = 100 nm. **(C)** Biochemical comparison of Mtb MVs produced by axenic Mtb cultures (“Ax. MVs”) or during Mtb infection of macrophages (“Inf. EVs”). The purification of infection-derived vesicles, which contain both Mtb MVs and exosomes, was performed as described (Athman et al., 2015). Western blotting was performed for Mtb lipoproteins (LprG, α1411c mAb; PhoS1, IT-23 mAb) and LAM (CS-35 mAb) which are markers of Mtb MVs. CD9 is a mammalian protein and marker for exosomes. **(D)** Mtb MVs induce DC maturation. Murine bone marrow-derived DCs were cultured for 24 h with no stimuli, 10 nM Pam_3_CSK_4_ (TLR2 agonist), or purified Mtb MVs. The expression levels of the antigen presentation molecules CD86, MHC-I, and MHC-II were determined by cell staining and flow cytometry. Values represent the specific MFI (mean fluorescence intensity) for CD11b+/CD11c+ cells. “PC” is used as an abbreviation for Pam_3_CSK_4_ in the figure only. Statistical significance is shown for treated DCs relative to the untreated control. Results are representative of 3 independent experiments. **(E)** Antigen presentation assay. Murine bone marrow-derived DCs were cultured for 24 h in the absence or presence of gel filtration-purified Mtb MVs. DCs were then cultured for an additional 24 h with the BB7 CD4+ T cell hybridoma. The ratio of DCs to T cells was 1:1 (100,000 cells each). IL-2 production for duplicate samples was measured using the murine IL-2 ELISA kit from R and D systems. Statistical significance is shown for IL-2 production in the absence or presence Mtb MVs. Results are representative of 3 independent experiments. **(F)** Mtb MVs inhibit IFNγ-mediated induction of MHC-II expression by uninfected macrophages. Murine bone marrow-derived macrophages were prepared as described (Athman et al., 2015) and treated with no stimuli, 50 ng/mL of Pam_3_CSK_4_, or 37–150 ng/mL Mtb MVs for 24 hr. (Note: the dose of MVs was based on total protein.) Cells were then activated with 2 ng/mL IFNγ for 24 h to induce MHC-II expression. Macrophages were stained using an anti-IA/IE mAb and then analyzed by flow cytometry. Statistical significance is shown for MV-treated macrophages relative to the control. Results are representative of 3 independent experiments. **(D–F)** Statistical analyses were performed using GraphPad Prism software and the two-tailed Students *t* test. Significance is indicated by asterisks (^*^p < 0.05; ^**^p < 0.01; ^***^p < 0.001). Ethics statement: All animal studies were approved by the Institutional Animal Care and Use Committee of Case Western Reserve University.

Our recent work has focused on how MVs released from Mtb-infected macrophages may modulate the functions of uninfected dendritic cells (DCs), macrophages, and T cells. To simplify the experimental model, we employed MVs that were produced by axenic Mtb cultures and purified. IZON qNano analysis (Figure 2A) and electron microscopy (Figure 2B) demonstrate that axenic culture-derived MVs are similar in size (80–250 nm) and morphology to those produced during Mtb infection of macrophages (Athman et al., 2015). The biochemical composition is also similar; axenic culture- and infection-derived MVs are enriched for Mtb lipoproteins (LprG, LpqH, Phos1) and glycolipids (LAM) (Figure 2C & data not shown).

The effect of Mtb MVs on DCs has yet to be addressed. First, we treated murine DCs with Mtb MVs or the synthetic TLR2 agonist Pam_3_CSK_4_ as a control and determined the effect on antigen presentation molecules. The expression of MHC-I, MHC-II, and CD86 were substantially increased, demonstrating that PAMPs in Mtb MVs induce DC maturation (Figure 2D). Several immunodominant antigens including Ag85b were identified as abundant proteins in Mtb MVs (Lee et al., 2015), so we next determined whether MVs could transfer T cell antigens to DCs. As shown in Figure 2E, DCs co-cultured with Mtb MVs and an Ag85b-specific CD4+ T cell hybridoma led to T cell activation as indicated by IL-2 production. Future studies are needed to explore the association of Ag85b with vesicles. Nonetheless, the release of MVs from Mtb-infected cells may provide a means for uninfected, lymph node DCs to acquire extracellular antigens, such as Ag85b, and prime CD4+ T cells (Figure 1B) (Srivastava and Ernst, 2014). Consistent with our findings, Mtb MVs administered to mice were capable of priming B and T cell responses, although the specific antigens were not identified in this study (Prados-Rosales et al., 2014a). In summary, the host may employ extracellular MVs as a readily accessible source of antigens to generate adaptive immune responses to Mtb.

In contrast, the extracellular release of Mtb MVs may impair effector T cell responses at the site of infection, and two mechanisms may be involved (Figure 1B). First, the treatment of uninfected macrophages with Mtb MVs inhibits IFNγ–induced MHC-II expression (Figure 2F). Thus, extracellular Mtb MVs may traffic to and subsequently impair the antigen presentation capacity of uninfected macrophages in the lung. The second mechanism is based on the observation that purified Mtb LAM inhibits TCR signaling and CD4+ T cell activation (Mahon et al., 2012; Sande et al., 2016) and that extracellular LAM is secreted via MVs from Mtb-infected cells (Athman et al., 2015). Our recent work demonstrates that Mtb MVs are enriched for LAM, inhibit T cell activation more potently than purified LAM, and induce T cell anergy (Athman et al., manuscript in revision). Thus, MVs that are released by Mtb-infected macrophages may traffic LAM to CD4+ T cells during infection to further suppress the adaptive immune response. In conclusion, Mtb MVs may induce innate and adaptive immune responses, but also promote immune evasion, and latent infection. As extracellular vesicle research progresses for other pathogens, it will be interesting to determine whether this is a general paradigm for the role of MVs in bacterial pathogenesis or a specialized case.

## Summary and perspectives

The past decade has seen tremendous growth in the field of MV research and has changed our viewpoint on fundamental aspects of microbial life. Although we are just beginning to understand how and why bacteria produce vesicles, MV secretion is likely a central mechanism for intercellular communication between prokaryotic and eukaryotic cells during infection. From an evolutionary perspective the production of MVs appears to be an ancient and conserved pathway that promotes the survival of bacteria, especially under conditions of environmental stress. In turn, the host immune system may have evolved to recognize MV-associated molecules as a readily accessible source of PAMPs and antigens to stimulate innate and adaptive immune responses, respectively. Thus, MV secretion by bacteria may contribute to virulence as well as immunity. However, studies to date have largely characterized MVs produced by axenic bacterial cultures. It is generally unknown if MVs are secreted during infection *in vitro* or *in vivo*, and if so, how MVs contribute to the host-pathogen interaction. Furthermore, individual studies have focused on the properties of bacterial MVs in a context that is either pathogen- or host-beneficial. To define the physiological roles of MVs, it will be necessary to assess the “net effect” of MV secretion on bacterial pathogenesis.

Moving forward, the determination of MV functions, such as those suggested for Mtb, awaits the development of new experimental models to study MVs in the context of infection. More specifically, mutant strains with impaired vesicle secretion are needed to determine the contribution of MVs to bacterial virulence and/or the host immune response. These tools are generally unavailable because of our limited understanding of MV synthesis pathways. Therefore, a high priority for the next phase of research is to elucidate the mechanisms of MV biogenesis for both gram-negative and gram-positive bacteria. Identification of the molecular machinery may also provide targets for new classes of antibiotics to treat bacterial infections or guide the design of MV-based vaccines.

## Author contributions

KJ and PW wrote the paper. KJ, YW, JA, and MB performed experiments.

## Funding

This work was supported by the NIH (R21 AI103443 to PW), the American Lung Association (Biomedical Research Grant to PW), and the Center for AIDS Research at Case Western Reserve University (NIH P30 AI036219).

### Conflict of interest statement

The authors declare that the research was conducted in the absence of any commercial or financial relationships that could be construed as a potential conflict of interest.
